# Hunger for iron: the alternative siderophore iron scavenging systems in highly virulent *Yersinia*

**DOI:** 10.3389/fcimb.2012.00151

**Published:** 2012-11-30

**Authors:** Alexander Rakin, Lukas Schneider, Olga Podladchikova

**Affiliations:** ^1^Phylogenomics of the Enteropathogenic Yersinia, Max von Pettenkofer-Institute, LMUMunich, Germany; ^2^Microbiology of Yersinia, Antiplague Research InstituteRostov-on-Don, Russia

**Keywords:** highly virulent Yersinia, iron acquisition, siderophores, pathogenomics, epidemic Yersinia

## Abstract

Low molecular weight siderophores are used by many living organisms to scavenge scarcely available ferric iron. Presence of at least a single siderophore-based iron acquisition system is usually acknowledged as a virulence-associated trait and a pre-requisite to become an efficient and successful pathogen. Currently, it is assumed that yersiniabactin (Ybt) is the solely functional endogenous siderophore iron uptake system in highly virulent *Yersinia* (*Yersinia pestis*, *Y. pseudotuberculosis*, and *Y. enterocolitica* biotype 1B). Genes responsible for biosynthesis, transport, and regulation of the yersiniabactin (*ybt*) production are clustered on a mobile genetic element, the High-Pathogenicity Island (HPI) that is responsible for broad dissemination of the *ybt* genes in *Enterobacteriaceae*. However, the *ybt* gene cluster is absent from nearly half of *Y. pseudotuberculosis* O3 isolates and epidemic *Y. pseudotuberculosis* O1 isolates responsible for the Far East Scarlet-like Fever. Several potential siderophore-mediated iron uptake gene clusters are documented in *Yersinia* genomes, however, neither of them have been proven to be functional. It has been suggested that at least two siderophores alternative to Ybt may operate in the highly virulent *Yersinia pestis*/*Y*. *pseudotuberculosis* group, and are referred to as pseudochelin (Pch) and yersiniachelin (Ych). Furthermore, most sporadic *Y. pseudotuberculosis* O1 strains possess gene clusters encoding all three iron scavenging systems. Thus, the Ybt system appears not to be the sole endogenous siderophore iron uptake system in the highly virulent yersiniae and may be efficiently substituted and/or supplemented by alternative iron siderophore scavenging systems.

Iron is one of the abundant elements on Earth, but it is hardly available to bacteria under aerobic conditions (10^−17^ M solubility limit for Fe(III) at pH ~7) and/or in mammalian hosts (10^−26^ M) (Otto et al., 1991). To obtain iron, bacteria have developed different strategies including one that utilizes low molecular weight siderophore molecules with high affinity to Fe^3+^ ions.

The human pathogenic *Yersinia* (*Y. pestis*, *Y. pseudotuberculosis*, and *Y. enterocolitica*) are ideal model organisms to study the impact of siderophore-mediated iron uptake systems on virulence as well as on the overall bacterial fitness and physiology. The pathogenic *Yersinia* can be divided into three main pathogroups, with *Y. pestis*, *Y. pseudotuberculosis*, and *Y. enterocolitica* biogroup 1B making up the highly virulent group (strains able to kill mice at low infection doses); low pathogenic *Y. enterocolitica* biogroups 2–5, and normally apathogenic *Y. enterocolitica* biogroup 1A (Carter, 1975; Heesemann, 1987). This grouping is based on the ability of bacteria of the high-pathogenic group to express an efficient siderophore yersiniabactin (Ybt) that plays a significant role in iron acquisition and murine pathogenicity (Carniel et al., 1987; Heesemann, 1987; Rakin et al., 1994). In contrast, two other pathogroups (low and apathogenic) do not produce Ybt.

Several other gene clusters with similarities to iron acquisition genes were uncovered by whole genome sequencing in *Y. pestis*/*Y*. *pseudotuberculosis* genomes, but their implication in iron uptake remains enigmatic (Forman et al., 2010). Therefore, Ybt is currently believed to be the exclusive endogenous siderophore of the highly-pathogenic *Yersinia*.

## Yersiniabactin-based iron acquisition system in highly virulent *yersinia*

**Figure 1:**
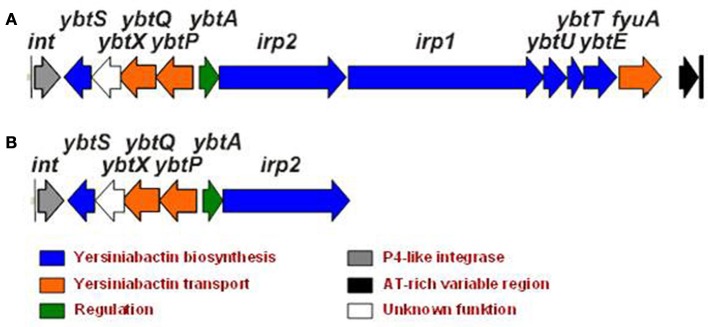
**Yersiniabactin gene cluster in *Y. pseudotuberculosis* O1 (A) and its truncated form in *Y. pseudotuberculosis* O3 (B)**.

The Ybt-siderophore system of *Yersinia* has been extensively studied during the last 20 years (for a recent review, see Perry and Fetherston, 2011). The structure of Ybt was determined and shown to contain phenolate, thiazoline, and thiazolidine rings and depicts high similarity to pyochelin produced by *Pseudomonas aeruginosa* and anquibactin of *Vibrio anguillarum*. The Ybt molecule is assembled by a typical strategy in which non-ribosomal peptide synthetase (NRPS) and polyketide synthetase (PKS) mediate biosynthesis. The strategy follows modular assembly of the siderophore from salicylate, a group from malonyl coenzyme A, three cysteine molecules and three methyl groups (Gehring et al., 1998). Six genes are involved in the Ybt synthesis and are designated *irp1*—*irp5*, *irp9* in *Y. enterocolitica*, and *irp1–2*, *ybtU, T, E, S*—in *Y. pestis* and *Y. pseudotuberculosis* (Figure 1). Irp9 (YbtS) directly converts chorismate into salicylate, the precursor of Ybt (Pelludat et al., 2003). Irp5 (YbtE) salicyl-AMP ligase transfers the activated salicylate to HMWP2 (encoded by *irp2*). HMWP2 possesses six predicted NRPS domains involved in initial cyclization and condensation reactions. Irp3 (YbtU) reduces the internal thiazoline ring to a thiozolidine structure while the first five domains of HMWP1 (encoded by *irp1*) switch from NRPS-type assembly line molecules to a PKS-strategy. Irp4 (YbtT) contains a thioesterase domain to remove aberrant structures from the enzymatic complex and displays an editing function together with terminal HMWP1 domains. The mechanism of Ybt secretion into the environment is currently unknown. Irp8 (YbtX in *Y. pestis)* was supposed to be involved in Ybt export (Fetherston et al., 1999), however, YbtX^−^ mutants are still able to secrete Ybt, so the function of this protein is still enigmatic. Fe-Ybt assimilation occurs through the outer membrane receptor FyuA (Psn in *Y. pestis*) and the inner cell membrane proteins Irp6 and Irp7 (YbtQ and YbtP in *Y. pestis*) (Fetherston et al., 1999; Brem et al., 2001; Perry and Fetherston, 2011). A periplasmic ferric Ybt transport protein has not been identified till now.

The biosynthetic and transport genes of Ybt are located on the High-Pathogenicity Island (HPI), a genomic mobile element that exhibits site-specific recombination activity of the P4-like integrase (Rakin et al., 1999; Carniel, 2001). The location of the Ybt-cluster on the mobile element accounts for its wide distribution in Enterobacteriaceae (Schubert et al., 2004; Antonenka et al., 2005).

Expression of the HPI genes is tightly regulated by host- and HPI-encoded factors. The Ybt biosynthetic and transport genes are repressed by iron loaded ferric uptake regulator (Fur)-protein and activated by the AraC-type YbtA transcriptional activator that also represses its own transcription (Fetherston et al., 1999; Anisimov et al., 2005). Besides, the functionality of the Ybt system is activated at the post-transcriptional level by P-pant transferase (YbtD, located outside the HPI) necessary for phosphopantetheinylation of NRP/PK- synthetases (Bobrov et al., 2002).

Inactivation of the Ybt biosynthetic and/or transport genes results in significant virulence attenuation of *Yersinia* (de Almeida et al., 1993; Heesemann et al., 1993; Rakin et al., 1994; Bearden et al., 1997; Pelludat et al., 1998; Brem et al., 2001). Ybt is the only known siderophore in *Y. enterocolitica*1B and its inactivation results in loss of the siderophore activity on chrome azurol S indicator plates (CAS-agar, used to detect a siderophore activity) (Schwyn and Neilands, 1987) and virulence attenuation. Also, the absence of Ybt in *Y. pestis* strains leads to the inability of bacteria to cause lethal infection by peripheral routes (Fetherston et al., 2010). The high significance of Ybt for establishing infection is supported by the comparison of the *ybt* genes expression in LB media and in the *in vivo* growth conditions. The *ybt* genes were up-regulated in the rat bubo after the subcutaneous *Y. pestis* inoculation (Sebbane et al., 2006) and in the lungs of mice after intranasal inoculation (Lathem et al., 2005; Liu et al., 2009). Interestingly, the *ybt* genes were not up-regulated in the mice spleen and liver in the pneumonic model of infection (Liu et al., 2009) and were down-regulated in the flea (Vadyvaloo et al., 2007, 2010). The increased expression of the *ybt* genes in mammalian hosts supports the importance of the Ybt siderophore for the *Yersinia* pathogenesis. Moreover, Ybt demonstrates a multifunctional character beyond iron binding and acquisition by its ability to protect bacteria from copper toxicity in uropathogenic *Escherichia coli* (Chaturvedi et al., 2012).

However, *Y. pseudotuberculosis* YPIII O3 type strain, widely used in animal experiments to address yersiniae virulence (Rosqvist and Wolf-Watz, 1986), completely lacks the Ybt iron acquisition system but demonstrates efficient iron binding on the CAS agar. In contrast, another group of the *Y. pseudotuberculosis* O3 strains possesses only a truncated form of the *ybt* gene cluster (Figure 1B) and shows no activity on the CAS agar. These strains also have lower virulence in animal models (Fukushima, 2003).

Furthermore, complete genome examination of epidemic *Y. pseudotuberculosis* IP31758 O1, an isolate that belongs to the group of strains responsible for the Far East Scarlet-like Fever (FESLF) in the Far East Russia and Japan (Gurleva et al., 1973; Eppinger et al., 2007), reveals the absence of the *ybt* gene cluster. Nevertheless, these strains demonstrate CAS activity and high virulence in humans and animals. Also the comparison of the genomes of IP32953 sporadic and IP31758 epidemic FESLF *Y. pseudotuberculosis* O1 strains favors the absence of the *ybt* genes from the epidemic strain. While the sporadic *Y. pseudotuberculosis* O1 strains contain a cluster identical to the *Y. pestis ybt*, the nosocomial “outbreak” FESLF strain, is *ybt-deficient*.

These results allude to the possibility that siderophore(s) alternative to Ybt are active in *Y. pestis/Y. pseudotuberculosis* highly virulent group.

## Pseudochelin—an alternative to the yersiniabactin siderophore

A potential siderophore producing locus with a high similarity of gene sequences and organization to the *ybt* gene cluster was discovered in all sequenced strains of *Y. pestis* and *Y. pseudotuberculosis* but not in *Y. enterocolitica* and has been designated the *Yersinia* non-ribosomal *p*eptide (*ynp*) (Perry and Fetherston, 2004; Forman et al., 2010). Because of these similarities, this cluster was initially annotated as a second HPI in *Y. pestis* CO92 (Parkhill et al., 2001). However, it lacks any elements of a mobile pathogenicity island such as the presence of an integrase encoding gene or recombination sites.

**Figure 2:**
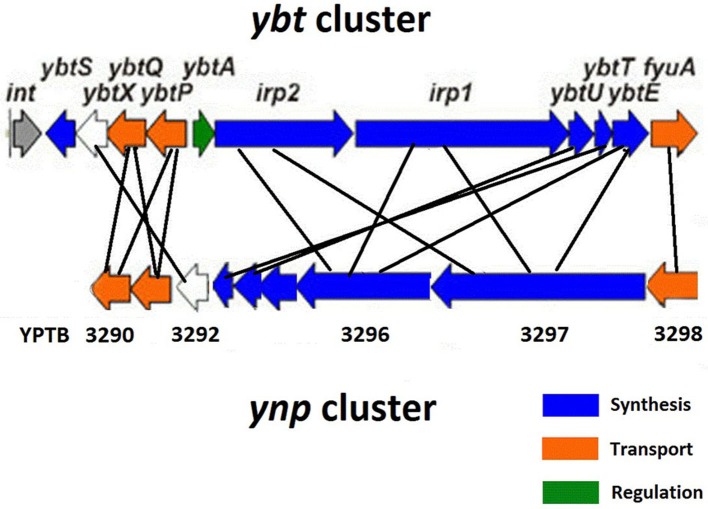
**Comparison of the *ybt* and *ynp* iron acquisition clusters in *Y. pseudotuberculosis* IP32953**.

In *Y. pseudotuberculosis* IP32953 the *ynp* locus (YPTB3290-3298) (Figure 2) contains putative NRPS and PKS siderophore assembly genes (YPTB3296-3297), ferri-siderophore transport genes (YPTB3290-3291), and a gene coding for the TonB-dependent outer membrane ferri-siderophore receptor (YPTB3298). Although the *ynp* locus shares many similarities with the *ybt* locus, the *ynp* locus appears to lack a salicylate synthase (*ybtS*) equivalent, suggesting that the putative siderophore may not use salicylate as a biosynthetic precursor. Based on the enzymatic domains encoded within the YPTB3296 and YPTB3297 genes, the structure of the putative Ynp siderophore was predicted to contain three thiazoline rings and have a molecular mass of 404.4/460.37 m/z with and without Fe^3+^, respectively (Forman et al., 2010).

Differential distribution of the *ybt* and *ynp* clusters in *Y. pseudotuberculosis* O1 is evidenced by the whole genome comparison of the epidemic *Y. pseudotuberculosis* IP31758 responsible for manifestation of FESLF and the typical “sporadic” *Y. pseudotuberculosis* IP32953 strains. While the latter has both clusters in its genome, the Far East strain lacks the *ybt* cluster and contains a frameshift mutation in the N-terminal region of the biosynthetic gene (YPTB3297). Nevertheless, it still demonstrates the CAS-positive phenotype suggesting that either some additional siderophore(s) mediate this activity or the truncated protein retains its function.

In contrast to the “sporadic” *Y. pseudotuberculosis* strains, the epidemic *Y. pestis* strains contain at least two mutations in the *ynp* locus. The NRPS (YPTB3297) is split by an IS*100* mobile element into two separate parts and contains a frameshift mutation in the C-terminal region (Forman et al., 2010). In the Microtus endemic *Y. pestis* 91001 strain, the YPTB3297 gene does not carry the IS*100* insertion, but still contains the frameshift mutation, as noted in the epidemic *Y. pestis* strains. In *Y. pestis* CO92 the *ynp* cluster is present in two separate chromosomal loci where YPO1011-1012 codes for the receptor (YPTB3298) and N-terminal part of YPTB3297 while YPO0770-0778 contains the remaining genes of the cluster (YPTB3290-3296). These observations have put the functionality of the *ynp* locus in *Y.pestis* into question (Forman et al., 2010). Thus, the *ynp* locus is likely specific to the sporadic *Y. pseudotuberculosis* strains and we propose that the encoded siderophore be called pseudochelin (Pch).

Classical models use iron limiting conditions *in vitro* to induce the expression of iron uptake systems and to identify iron regulated genes. The expression of the *ynp* genes has been supported by both *in vivo* and *in vitro* studies using microarray and quantitative real time PCR. Since a Fur-like binding region is located 184 bp upstream of the putative *ynp* receptor transcriptional start site in *Y. pestis*, expression of this locus is likely Fur-dependent and activated under iron limited conditions (Han et al., 2007; Gao et al., 2008). Several studies indicate that simulation of low iron concentrations *in vitro* results in up-regulation of the putative *ynp* receptor gene (YPTB3298) in *Y. pestis* and *Y. pseudotuberculosis* (Han et al., 2007; Gao et al., 2008; Rosso et al., 2008). When *Y. pestis* was grown in human plasma the putative *ynp* receptor and the biosynthetic gene (YPTB3297) were up-regulated relative to growth in LB media (Chauvaux et al., 2007). The *Y. pestis* murine pneumonic infection model showed nearly equal expression levels for the *fyuA* and *ynp* putative siderophore receptor genes 48 h post-intranasal infection providing evidence that pseudochelin is an early infection virulence factor (Lathem et al., 2005). Expression analysis in the rat bubonic infection model complements these findings by confirming up-regulation of the receptor and biosynthetic genes (YPTB3298 and YPTB3296) *in vivo* as compared to the expression levels found in LB media (Sebbane et al., 2006; Vadyvaloo et al., 2010). A proteomic analysis revealed that the putative Ynp receptor (homolog of YPTB3298) was present at higher concentrations in the outer membrane extracts of *Y. pestis* KIM6 at 26°C vs. 37°C when cells were grown in complete PMH2 media (Pieper et al., 2009).

Due to their many similarities, co-operation and crosstalk between the *ynp* and *ybt* gene clusters has been hypothesized. Since, the *ynp* operon lacks P-pant, a necessary transferase component used to commence the NRPS/PKS system, it has been suggested that the Ybt siderophore assembly machinery, namely YbtD, could compensate by performing this function (Forman et al., 2010). It has also been proposed that the *Y. pestis* KIM6 Ybt-negative strain produces Ybt-like molecules that are present in culture supernatants and are capable of *ybt* transcriptional activation (Miller et al., 2010). Since this data suggest that compounds produced outside of the *ybt* gene cluster can activate the *ybt* genes, yersiniabactin-like molecules are present in the media and the predicted structure of the Pch siderophore shares similarities with Ybt, it is likely that the products of the *ynp* locus participate in this crosstalk. Whether the aberrant Ybt-like molecules are produced by the *ynp* operon, has not yet been experimentally established, however, this evidence provides insight into their ability to influence other siderophore producing systems (Miller et al., 2010).

## Yersiniachelin-a second alternative to the yersiniabactin in pathogenic *yersinia*

Another *Yersinia* siderophore was found as a component of the autoagglutination factor (AF) purified from the surface of the Ybt-negative strain *Y. pestis* EV76 (Podladchikova and Rykova, 2006). AF appeared to be a complex antigen composed of the 17,485-kDa protein and an iron-loaded low molecular weight component that demonstrated siderophore activity. The AF protein was identified as Ypo0502 (Podladchikova et al., 2011), the Hcp (hemolysin coregulated protein)-like component of an extracellular apparatus of the type six secretion system (T6SS) recently characterized in gram-negative bacteria (for review, see Records, 2011). Hcp monomers are known to self-assemble on the cell surface into pilus-like filaments through which T6SS-associated proteins and effectors are transported. The physiological consequence of the siderophore interaction with the Hcp-like protein on the *Y. pestis* cell surface awaits further investigation.

The AF-derived siderophore was considered to be a novel *Yersinia* siderophore (Podladchikova et al., 2012), as it was purified from the strain (*Y.pestis* EV76), which has no genes coding for Ybt synthesis. Chemical analysis of the novel siderophore, designated yersiniachelin (Ych), revealed that it contained hydroxamate groups. In available genome sequences of *Y. pestis*, two gene clusters are able to code for hydroxamate siderophores. The first, homologous to the aerobactin siderophore cluster, carries several mutations in biosynthetic genes and was shown to be non-functional in *Y. pestis* (Forman et al., 2007). The other cluster (YPO1528-1538 in *Y. pestis* CO92) designated *ysu* (*Yersinia s*iderophore *u*ptake), which contains hydroxamate siderophore synthesis and transport genes, has no visible mutations and thus is predicted to encode for the biosynthesis of a functional siderophore (Perry and Fetherston, 2004; Forman et al., 2010). According to bioinformatic data the *ysu*-cluster is present in all sequenced *Y. pestis* and *Y. pseudotuberculosis* strains but not in *Y. enterocolitica*.

**Figure 3:**
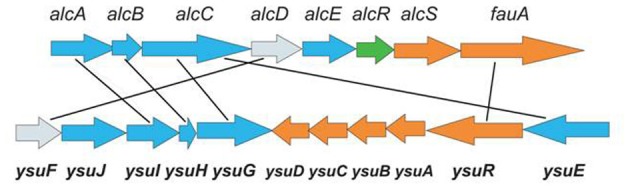
**Comparison of the *alc*-cluster encoding the production of the alcaligin siderophore in *Bordetella* spp with the *ysu*-cluster encoding the production of the yersiniachelin siderophore in *Y. pestis***. Siderophore biosynthetic genes are shown in blue, transport genes—in orange, regulatory gene—in green, and ferric reductase genes with unknown function—in grey.

The involvement of the *ysu* locus in Ych production was confirmed by the analysis of a *Y. pestis* EV76 knock-out mutant in which three biosynthetic genes of the putative hydroxamate siderophore (*ysu IHG)* were deleted (Podladchikova et al., 2012). In contrast to the parent strain, the mutant did not produce Ych. These results provide evidence that Ych is encoded by the *ysu* locus, so we propose that *ysu* may stand for *y*ersiniachelin *s*ynthesis and *u*tilization. The *ysu*-cluster is similar to the *alc*-cluster (Figure 3) coding for the hydroxamate siderophore alcaligin (Alc) produced by *Bordetella spp* and important for the multiplication *in vivo* and virulence of bacteria (Brickman et al., 2008, 2011; Brickman and Armstrong, 2009). The Alc siderophore is well-studied and known to be assembled from diamine precursors by a process-independent of the NRPS-pathway and performed by a number of enzymes encoded by the *alc*-cluster on the *Bordetella* chromosome. The Alc biosynthesis begins with ornithine decarboxylation by Odc (encoded outside the *alc*-cluster) followed by a N-hydroxylation by AlcA and N-acetylation of the hydroxylamine group by AlcB. Then the product is C-hydroxylated by AlcE and undergoes dimerization and macrocyclization performed by AlcC.

Comparison of the *ysu* and *alc* clusters shows similarities in DNA and protein sequences of a number of genes and differences in genetic organization and content. The homology is obvious in the four siderophore biosynthetic genes (*ysu*GHIJE and *odc*, *alc*ABC), the ferri-siderophore outer membrane receptors (*ysu*R and fauA), as well as the ferric iron reductase genes (*ysuF* and *alc*D) whose role in Alc siderophore function is currently unknown. Though homologous in the siderophore biosynthetic and receptor genes, the two clusters have several apparent differences. First, a homolog of the *alc*E biosynthetic gene coding for an iron-sulfur protein involved in C-hydroxylation of precursor molecules during Alc biosynthesis, is absent from the *ysu*-cluster and *Y. pestis* genome. This suggests that the structure of Ych may be slightly different from Alc. Nevertheless, due to the obvious homology of the four siderophore biosynthetic genes several possible structures of the *ysu*-encoded putative macrocyclic hydroxamate siderophore have recently been proposed (Forman et al., 2010). Second, in the *ysu*-cluster there is no homolog of the alcS gene which codes for the major facilitator superfamily (MFS)-like protein shown to be involved in the Alc siderophore efflux by *Bordetella* cells (Brickman and Armstrong, 2005). Currently the mechanism of Ych export from bacteria is obscure. Third, the *ysu*-cluster carries genes (*ysu*ABCD) coding for ferri-siderophore transporters through the periplasm and cytoplasmic membrane which are absent from the *alc* cluster. This step of the siderophore-based iron acquisition in *Bordetella* has been suggested to be performed by an alternative mechanism (Brickman et al., 2011). The FbpA ABC-transporter, (a periplasmic iron-binding protein encoded outside the *alc* cluster) was shown to transport iron liberated in the periplasm from multiple structurally distinct siderophores through the cytoplasmic membrane. Fourth, the *ysu*-cluster lacks the *alc*R homolog coding for the AraC-type transcriptional regulator AlcR which, in complex with ferri-Alc, activates the expression of the siderophore biosynthesis and transport genes by *Bordetella* (Brickman and Armstrong, 2009).

The expression of the *ysu* genes by *Y. pestis* is supported by multiple microarray studies which have been applied to various *in vitro* growth conditions, *ex vivo* growth in human plasma and macrophages, as well as *in vivo* animal infection models. The transcriptomic data on different *Y. pestis* strains grown *in vitro* indicate that the *ysu* genes are transcribed in culture broth independent of temperature (Han et al., 2004; Motin et al., 2004) and are repressed in the presence of iron by the Fur protein and up-regulated in iron-deficient conditions (Zhou et al., 2006; Gao et al., 2008). Comparative transcriptome analysis of *Y. pestis* in different *in vitro* conditions (Han et al., 2005, 2007) revealed additional signals sensed by the *ysu*-locus genes which were upregulated during hyperosmotic and high-salinity stress in OmpR-dependent manner. This data infers that OmpR might play the role of a transcriptional activator for *ysu* gene expression, the function which is performed by AlcR for the *alc* genes expression.

The *ex vivo* transcriptome analysis of *Y. pestis* and *Y. pseudotuberculosis* grown in human plasma (Chauvaux et al., 2007; Rosso et al., 2008) showed that the *ysu* genes were up-regulated in plasma in both species. In contrast, no difference in expression was observed in Ybt-negative *Y. pestis* KIM5Δpgm inside J774.1 macrophage-like cells (Fukuto et al., 2010). During intracellular multiplication a number of *Y. pestis* genes involved in iron acquisition (*hmu, yfe*, and *yiu*) were down-regulated suggesting that in macrophages *Y. pestis* is not starved for iron.

The *ysu* genes were not expressed *in vivo* in the flea vector (Vadyvaloo et al., 2007, 2010) and the level of their expression in mice lungs, spleen and liver during primary pneumonic plague did not differ from the level detected in LB medium (Lathem et al., 2005; Liu et al., 2009). However, the *ysu*, *ybt* and *yfe* genes were upregulated in the bubo of *s.c*. infected rats (Sebbane et al., 2006). This result is consistent with the observation that the *ysu* genes are induced not only by the iron starvation but also by the high osmolarity signal and point to a possible involvement of Ych in the initial stages of infection after *s.c*. challenge.

The production of the *ysu* encoded proteins by *Y. pestis* was confirmed by proteomic analysis (Pieper et al., 2008, 2009). Two *ysu* encoded proteins, the biosynthetic protein YsuI (AlcA homolog) and siderophore receptor YsuR (FauA homolog), were identified in the membrane proteome of *Y. pestis* KIM6 grown to stationary phase at 26°C in chemically defined PMH2 medium. The YsuR protein associated with the outer membrane was increased in abundance in iron-deficient conditions (Pieper et al., 2010). Moreover, in iron-starved cells the amount of the siderophore biosynthetic protein YsuG (AlcC homolog) was also increased in the periplasmic cell fraction. Thus, the last steps of the Ych synthesis might take place in the periplasm after the transport of the siderophore precursor through the cytoplasmic membrane.

Thus, in highly virulent *Yersinia* the expression of the third siderophore system encoded by the *ysu* locus is substantiated by the mutagenesis of the *ysu* genes, transcriptomic and proteomic data, as well as by the purification of the Ych siderophore from the *ybt*-negative *Y. pestis* strain. The up-regulation of the *ysu* genes in iron-deplete conditions *in vitro* point to the possible involvement of Ych in iron acquisition. However, the exposure of Ych associated with the Hcp-like protein YPO502 on the bacterial surface in iron-replete conditions and the up-regulation of its biosynthetic genes by high osmotic and salt stress suggest an additional role for Ych in *Y. pestis* physiology.

## Summary

Like most pathogenic bacteria, highly-virulent *Yersinia pestis /Y. pseudotuberculosis* possess several alternative endogenous siderophore-mediated iron acquisition systems. A complete functional understanding of these novel siderophore systems is currently lacking in the literature and future studies should focus on parsing out the impact these systems have on bacterial physiology.

### Conflict of interest statement

The authors declare that the research was conducted in the absence of any commercial or financial relationships that could be construed as a potential conflict of interest.
